# Psychometric properties of self-reported measures of health-related quality of life in people living with HIV: a systematic review

**DOI:** 10.1186/s12955-021-01910-w

**Published:** 2022-01-10

**Authors:** Huan Wen, Zhongfang Yang, Zheng Zhu, Shuyu Han, Lin Zhang, Yan Hu

**Affiliations:** 1 Fudan University School of Public Health, Shanghai, China; 2 School of Nursing, Fudan University, Shanghai, China; 3 Fudan University Centre for Evidence-Based Nursing: A Joanna Briggs Institute Centre of Excellence, 305 Fenglin Rd, Shanghai, 200032 China; 4 Peking University School of Nursing, Beijing, China; 5 Shanghai Public Health Clinical Center, Shanghai, China

**Keywords:** HIV, AIDS, Quality of life, PROM, Systematic review

## Abstract

**Objective:**

To identify and assess the psychometric properties of patient-reported outcome measures (PROMs) of health-related quality of life (HRQoL) in people living with HIV (PLWH).

**Methods:**

Nine databases were searched from January 1996 to October 2020. Methodological quality was assessed by using the Consensus-based Standards for the Selection of Health Measurement Instruments (COSMIN) Risk of Bias Checklist. We used the COSMIN criteria to summarize and rate the psychometric properties of each PROM. A modified Grading, Recommendations, Assessment, Development, and Evaluation (GRADE) system was used to assess the certainty of evidence.

**Results:**

Sixty-nine studies reported on the psychometric properties of 30 identified instruments. All studies were considered to have adequate methodological quality in terms of content validity, construct validity, and internal consistency. Limited information was retrieved on cross-cultural validity, criterion validity, reliability, hypothesis testing, and responsiveness. High-quality evidence on psychometric properties was provided for the Medical Outcomes Study HIV Health Survey (MOS-HIV), the brief version of the World Health Organization's Quality of Life Instrument in HIV Infection (WHOQoL-HIV-BREF), 36-Item Short Form Survey (SF-36), Multidimensional Quality of Life Questionnaire for Persons with HIV/AIDS (MQoL-HIV), and WHOQoL-HIV.

**Conclusions:**

The findings from the included studies highlighted that among HIV-specific and generic HRQoL PROMs, MOS-HIV, WHOQoL-HIV-BREF, SF-36, MQoL-HIV, and WHOQoL-HIV are strongly recommended to evaluate HRQoL in PLWH in research and clinics based on the specific aims of assessments and the response burden for participants.

**Supplementary Information:**

The online version contains supplementary material available at 10.1186/s12955-021-01910-w.

## Introduction

With the introduction of antiretroviral therapy (ART), the life expectancy of PLWH has been prolonged. However, HIV, ART, infectious diseases, comorbidities, and premature aging pose challenges to the health-related quality of life (HRQoL) of PLWH. HRQoL can be defined as one’s perceived functioning in the physical, emotional, psychological, and social domains of health [1]. Alternatively, HRQoL was defined by Torrance as a concept incorporating factors that are part of an individual’s health [2]. HRQoL is currently regarded as a health aspect of quality of life (QoL); nonhealth aspects, including economic and political circumstances, are not included in HRQoL. Achieving a high level of HRQoL has become an important issue and a component of HIV/AIDS care [3]. In 2016, Lazarus and colleagues proposed adding a fourth “90” to the existing “90–90–90” target [4, 5]. The fourth 90% target is 90% of PLWH with viral load suppression to have good HRQoL. According to the World Health Organization's 90–90–90–90 goals, improving the HRQoL of PLWH is the ultimate goal of HIV/AIDS treatment and care [6, 7]. However, which measures are the most suitable is still under debate.

Many HIV-specific and generic HRQoL patient-reported outcome measures have been validated in different contexts. As one of the earliest HIV-specific HRQoL PROMs, MOS-HIV is the most commonly used measure [8]. The MOS-HIV consists of 35 items and 10 dimensions, including general health perceptions, physical functioning, role functioning, pain, social functioning, mental health, energy, health distress, cognitive functioning, and overall self-rated quality of life. In addition to MOS-HIV, other HIV-specific HRQoL PROMs are also widely used, including the WHOQoL-HIV-BREF [9], Multidimensional Quality of Life Questionnaire for Persons with HIV/AIDS (MQoL-HIV) [10], HIV Disease Quality of Life 31-Item Instrument (HIV-QL31) [11], and Patient-Reported Outcomes Quality of Life–HIV instrument (PROQoL-HIV) [12]. Additionally, validated subscales or scales with over 40 items, such as the World Health Organization Quality of Life-HIV (WHOQoL-HIV) [13], HIV Overview of Problems Evaluation Scale (HOPES) [14], Functional Assessment of HIV Infection (FAHI) [15], HIV/AIDS Targeted Quality of Life (HAT-QoL) [16], and HIV/AIDS Quality of Life Questionnaire (HIV/AIDSQoL) [17], are also used to evaluate HRQoL. In addition to HIV-specific PROMs, some generic PROMs, including the Short Form Health Survey (SF-12, SF-36) [18, 19], EuroQol—5 Dimensions (EQ-5D) [20, 21], World Health Organization Quality of Life assessment (WHOQoL) [22], Medical Outcomes Study Health Survey (MOS) [23], Missoula-Vitas Quality-of-Life Index (MVQOLI) [24], Patient-Reported Outcomes Measurement Information System (PROMIS) [25], Health Assessment Questionnaire Disability Index (HAQ-DI) [26], Quality of Well-Being scale (QWB) [27], and Health Utility Index 3 (HUI3) [28], have been validated and used in the PLWH population globally. The advantage of using generic HRQoL PROMs is that researchers can directly compare the results with those of other groups based on the same problem without standardizing the data. However, for PLWH, generic PROMs may not be as sensitive as specific PROMs assessing HIV-specific dimensions of HRQoL regarding stigma, relationship issues, and comorbidities [29].

A preliminary literature search was conducted in PubMed, PsycINFO (EBSCO), Cochrane Library (Wiley) and JBI (Ovid), and many reviews on measures of HRQoL were found. Cooper et al. [29] briefly summarized PROMs with fewer than 40 items for measuring HRQoL in PLWH and found that the MOS-HIV was the most well-established measure. The WHOQoL-HIV-BREF and PROQoL-HIV were considered to have good psychometric properties and to potentially have more relevance to PLWH than other PROMs. However, the study included only instruments that can be completed within 10 min or that have fewer than 40 items. Additionally, the assessment process of psychometric properties was not systematic enough to provide a concrete conclusion. Clayson et al. [30] conducted reviews with similar aims but in a specific context (in clinical trials and in sub-Saharan Africa) in 2006 and 2010, respectively. Gakhar et al. conducted a nonsystematic review of the literature on quality of life assessment after ART in developed countries in 2013 [31].

However, previous systematic reviews have mainly focused on the content of HRQoL PROMs and have not reported their psychometric properties, which has made it difficult for healthcare professionals to select one of the existing PROMs to evaluate HRQoL in research and clinical practice [29–31]. Accurate and reliable PROMs are a prerequisite for obtaining robust results. It is critical to choose an acceptable PROM with good psychometric properties [32]. Therefore, to obtain reliable evidence regarding the psychometric properties of HRQoL PROMs, we conducted a systematic review to identify and assess the psychometric properties of PROMs of HRQoL in PLWH. This conclusion may provide a scientific basis for researchers to choose PROMs for future scientific research and clinical practice measuring HRQoL in PLWH.

## Methods

### Aims and design

The aim of this study was to identify and assess the psychometric properties of PROMs of HRQoL in PLWH. This systematic review was performed with the guidance of the Joanna Briggs Institute (JBI) methodology for systematic review of psychometric properties and the Preferred Reporting Items for Systematic Reviews and Meta-Analyses (Additional file 1: PRISMA) statement. The protocol of our review was published in *JBI Evidence Synthesis* [33].

### Search strategy

We conducted a three-step search. First, a limited search was conducted in PubMed to develop search strategies tailored to each database. Second, researchers implemented the search strategies in PubMed, MEDLINE (Ovid), EMBASE (Ovid), CINAHL (EBSCO), Web of Science, ProQuest Dissertations and Theses, Cochrane Library (Wiley), CNKI, and WanFang. The databases were searched for published studies from 1st January 1996 to 1st May 2020. We set the start point 1996 because ART was first used in 1996. Google Scholar and Baidu Scholar were searched for gray literature. We used MeSH terms ([“HIV” OR “Acquired Immunodeficiency Syndrome”] AND “Quality of Life”) combined with ([HIV OR AIDS OR “acquired immunodeficiency syndrome”] AND “quality of life” AND “COSMIN search filter”). Additional file 2: Appendix I lists the search strategies used for all databases. Finally, we manually reviewed all references included during the supplemental searches.

### Inclusion and exclusion criteria

The inclusion criteria were as follows: (1) studies that targeted HIV-positive adults (≥ 18 years old); (2) studies of any types of self-reported measures, including but not limited to, self-management questionnaires that aimed to measure HRQoL among PLWH; (3) validation studies or studies that aimed to develop PROMs or assess one or more measurement properties; and (4) studies published in either English or Chinese. The exclusion criteria included the following: (1) studies that aimed to validate measures assessing only a certain domain of HRQoL related to specific comorbidities or treatment side effects and (2) studies that provided indirect evidence of psychometric properties (e.g., comparing one PROM with another instrument).

### Study screening and selection

We imported all references identified in the search into Endnote X8 (Clarivate Analytics, PA, USA). After the removal of duplicates, two researchers (HW & ZY) screened the titles, abstracts, and full texts independently to assess whether the studies met the eligibility criteria. Any discrepancies were resolved by the third researcher (ZZ). The reasons for exclusion of studies at the full-text screening stage were recorded.

### Quality appraisal

Two reviewers (HW & ZY) assessed the included studies independently by using the COSMIN Risk of Bias Checklist. When there were discrepancies, a third reviewer (ZZ) was included to resolve them. The COSMIN Risk of Bias Checklist consisted of 10 domains (38 items), including PROM development, content validity, structural validity, hypothesis testing of construct validity, cross-cultural validity/measurement invariance, criterion validity, internal consistency, measurement error, test-test reliability, and responsiveness. The options for each item included “very good”, “adequate”, “doubtful”, and “inadequate quality”. The methodological quality of the study was based on the worst score counts.

### Data extraction and synthesis

Two researchers (HW & ZY) independently extracted information, including the author, publication year, country/language, study design, target population, sample size, measurement domains, number of items, and total score range. The main findings regarding psychological properties included construct validity, internal consistency, cross-cultural validity/translation, criterion validity, and reliability. Any discrepancies were discussed between the two researchers.

We used the COSMIN criteria to summarize and rate the psychometric properties of each study regarding structural validity, internal consistency, reliability, measurement error, hypothesis testing for construct validity, cross-cultural validity/measurement invariance, criterion validity, and responsiveness. Each measurement property was rated as sufficient (+), insufficient (−), or indeterminate (?). When data were synthesized and the ratings of each study were consistent, the overall rating of the measurement property was rated as sufficient (+) and insufficient (−). If the ratings of each study were all sufficient (+), the overall rating of the measurement property was rated as sufficient (+). If the ratings of each study were all insufficient (−), the overall rating of the measurement property was rated as insufficient (−). We used narrative synthesis to synthesize the data for each measurement property. If the ratings of each study were inconsistent, we explored possible explanations (e.g., different languages). If the explanation was reasonable, we provided ratings by subgroup. If the explanation was unreasonable, the overall rating of the measurement property was rated as inconsistent (±). If there was no information to support the rating, the overall rating was rated as uncertain (?).

### Assessment of the certainty of the evidence

We used a modified Grading of Recommendations, Assessment, Development, and Evaluation (GRADE) system to assess the certainty of the evidence. Each piece of evidence was graded for risk of bias, inconsistency, imprecision, and indirectness. Four reviewers (HW, ZY, ZZ, and SH) graded each measurement property and each PROM separately. Discrepancies were resolved by the fifth reviewer (YH). Based on the methodological quality of each psychometric property, four reviewers finally classified the instruments as strongly recommended, weakly recommended and not recommended according to the modified GRADE system. The classification results were verified by all authors.

## Results

### Literature search

**Fig. 1:**
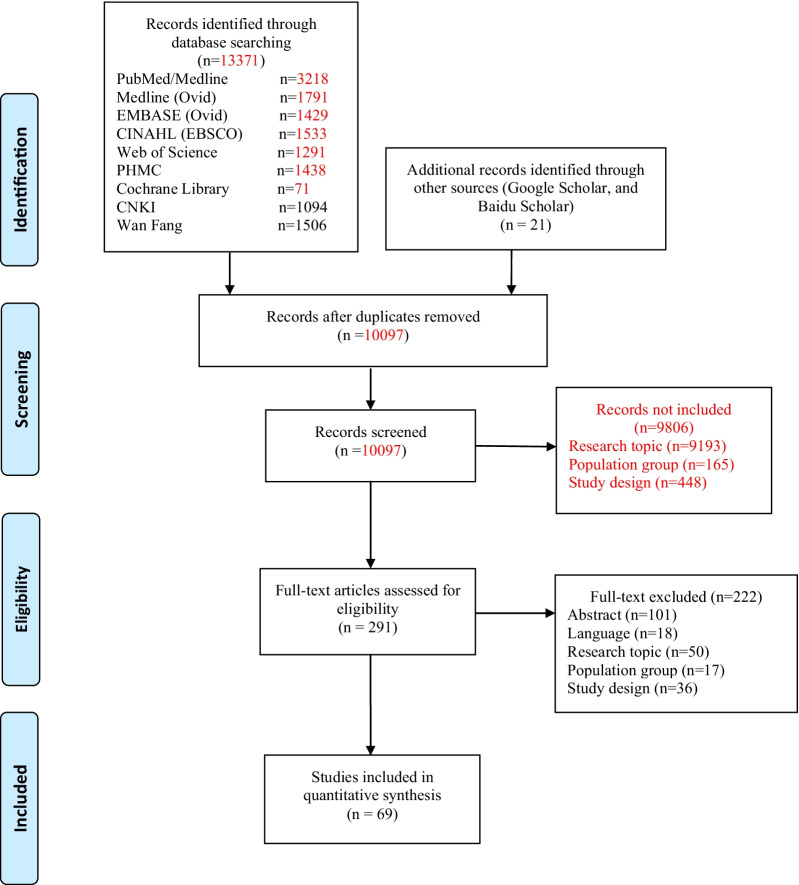
Flowchart of the identification and selection of studies

The literature screening and selection process is shown in Fig. 1. In the initial search, a total of 13,371 articles were identified in the databases. Twenty-one articles were found through additional supplementary searches. After the removal of duplicates, a total of 10,097 articles were retained, and 10,028 articles were deleted after the review of the titles, abstracts, and full text. We finally included 69 articles [9–28, 34–82]. A total of 30 PROMs were investigated in the included studies.

### Study description

Among the 69 included articles, 54 were in English, and 15 were in Chinese; the articles were published from 1996 to 2019. A description of the studies is shown in Table 1. All the included studies were cross-sectional studies. Twenty studies were conducted in China [17, 22, 36–40, 57–62, 74, 77–82], fourteen in the United States [15, 16, 21, 25–27, 35, 42, 64, 65, 67, 72, 73, 75], three in Uganda [24, 41, 46], three in Italy [44, 49, 69], two in Australia [70, 71], two in Vietnam [20, 55], two in Portugal [52, 75], and two in Canada [28, 66]. A total of 28,480 participants were included, with sample sizes ranging from 50 to 1923 [9–28, 34–82]. One study was conducted with adult males [35]. Four studies were conducted with HIV-positive women [41, 42, 65, 66]. One study was conducted with HIV-infected patients aged 50 years and older [52], and two studies were conducted with people with advanced AIDS [24, 28]. One study involved transgender male, transgender female, and genderqueer individuals [25]. One study was conducted in patients with HIV-related opportunistic infections [47].

**Table 1:** Overview of the included studies

References	PROM	Country	PROM language	Study design	Target population	Sample size	Year of development/validation	Measurement domain	Number of items	Total score range
Akinboro et al. [63]	WHOQOL-BREF *Nigerian version*	Nigeria	Nigerian	Cross-sectional study	PLWH, mean age: 38.5 ± 9.7 Male: 144, Female: 347	491	Between July 2010 and January 2011	Physical health; psychological health; level of independence; social relationships; environmental health; spirituality, religion and personal beliefs	31	NR
Ahmed et al. [56]	WHOQOL-HIV-BREF *Urdu version*	Pakistan	Urdu	Cross-sectional study	PLWH, age: < 25 years: 30; 25–50 years: 104, > 50 years: 48 Male: 134, Female: 48	182	NR	Physical health; psychological health; level of independence; social relationships; environmental health; spirituality, religion and personal beliefs	31	(− 2)− 2
Brown et al. [70]	PozQoL	Australia	English	Cross-sectional study	PLWH, age: 18–34: 34, 35–49: 157 50–64: 208, 65 + : 66 Male: 378, Female: 14 Participants who either did not answer the question or indicated some other gender: 73	465	Between March 22 and May 31, 2017	Health concerns, psychological, social, functional	64	1–5
Bucciardini et al. [69]	ISSQoL *Italian version*	Italy	Italian	Cross-sectional study	PLWH, Female: 118 (35.5) Male: 202 (60.8) Missing information: 12 (3.6), age Mean ± SD: 40.0 ± 7.3	332	NR	Satisfaction with quality of life, physical well-being, role well-being, depression and anxiety, energy and vitality, health distress, cognitive functioning, social functioning, sexual life, social support, interaction with medical staff, treatment, impact, body changes, life planning, motherhood/fatherhood	62	0–100
Connell and Skevington [51]	WHOQOL-HIV-BREF	Australia, Brazil, Bangalore, New Delhi, Thailand, Zimbabwe, Italy, Ukraine	Brazilian, Bangalore, New Delhi, Thai, Zimbabwean, Italian, Ukrainian	Cross-sectional study	PLWH, mean age: 33.4 ± 9.8 Male: 1271, Female: 652	1923	NR	Physical health; psychological health; level of independence; social relationships; environmental health; spirituality, religion, and personal beliefs	31	4–20
De Boer et al. [14]	HOPES *Dutch and English versions*	Netherlands	Dutch, English	Cross-sectional study	PLWH Mean age: 38 ± 7.8 Male: 99, Female: 7	106	NR	Physical, psychosocial, medical interaction, sexuality, partner	142	NR
Duracinsky et al. [12]	PROQoL-HIV *English, Brazilian, Cambodia, Chinese, French, Senegalese, and Thai versions*	Australia, Brazil, Cambodia, China, France, Senegal, Thailand, USA	English, Brazilian, Cambodia, Chinese, French, Senegalese, Thai	Cross-sectional study	PLWH, median age: 41 Male: 506, Female: 285	791	Between July and December 2008	General health perception, social, relationships, emotions, energy/fatigue, sleep, cognitive functioning, physical and daily activity, coping, future, symptoms, and treatment	67	0–4
Fang et al. [22]	WHOQOL *Chinese version*	China	Chinese	Cross-sectional study	PLWH, age (years) ≤ 30: 34, 31–40: 42 > 40: 24 Male: 96 Female: 40	136	NR	Physical, psychosocial, social, environment	26	1–20
Herrmann et al. [71]	PROQoL-HIV	Australia	English	Cross-sectional study	PLWH, mean age: 46 (37–53.8) Male: 87, Female: 15	102	NR	Physical health and symptoms, emotional distress, health concerns, body change, intimate relationships, social relationships, stigma	31	1–100
Holmes and Shea [16]	HAT-QoL	US	English	Cross-sectional study	PLWH Mean age: 37.8 (8.9) Male: 78, Female: 28	106	Between January and March 1996	Overall function (physical function, role function and social function), sexual function, disclosure worries, health worries, financial worries, HIV mastery, life satisfaction, medication concerns, provider trust	42	0–100
Holmes and Shea [76]	HAT-QoL	US	English	Cross-sectional study	PLWH Mean age: 37.8 (8.6) Male: 173, Female: 42	215	Between May and August, 1996	Overall function, sexual function, disclosure worries, health worries, financial worries, HIV mastery, life satisfaction, medication concerns, provider trust	42	NR
Hsiung et al. [57]	WHOQOL-HIV-BREF *Chinese version*	China	Chinese	Cross-sectional study	PLWH in Taiwan Age: 36.3 (10.1) Male: 646, Female: 28	674	NR	General health; physical health; level of independence; psychological health; spirituality, religion and personal beliefs; social relations; environmental health	31	4–20
Hughes et al. [35]	MOS-HIV-34	US	English	Cross-sectional study	Adult males, HIV-infected Mean age: 35.3	100	Between September 14, 1992, and March 16, 1993	Overall health, pain, physical function, role function, social function, cognitive function, mental health, energy/fatigue, health distress, quality of life, health transition	34	NR
Kaplan et al. [26]	QWB scale	US	English	Cross-sectional study	PLWH Male: 400, Female: 114	514	NR	NR	NR	NR
Kemmler et al. [10]	MQoL-HIV *German version*	Germany	German	Cross-sectional study	PLWH, mean age: 37.8 ± 9.5 Male: 118, Female: 89	207	NR	Physical, emotional, cognitive, social and financial aspects, sexual functioning, medical care	40	0–100
Kohli et al. [23]	MOS *Indic version*	India	Indic	Cross-sectional study	PLWH, age: < 20:1, 20–29:28 30–39:52, 40–49:13, ≥ 50: 6, Male: 66, Female: 34	100	Between February 2002 and March 2003	Physical health, work and earnings, daily routine, social activities, cognitive function, feelings and emotions, pain, sleep, food and appetite, sexual life	29	0–100
Kusterer et al. [19]	SF-36v2 *Brazilian-Portuguese version*	Brazil	Brazilian-Portuguese	Cross-sectional study	PLWH, mean age: 44 ± 11.3 Male: 219 (55.9) Female: 173 (44.1)	392	NR	Physical functioning, role-physical, bodily pain, general health, vitality, social functioning, role-emotional, mental health	36	NR
Lau et al. [36]	MOS-HIV *Chinese version*	China	Chinese	Cross-sectional study	PLWH, age: (mean = 38.38, SD = 9.75) Male: 213 Female: 29	242	Between January and April 2000	General health, physical function, role function, social function, cognitive function, pain, mental health, energy/fatigue, health distress, quality of life	35	NR
Leplège et al. [11]	HIV-QL31 *French version*	France	French	Cross-sectional study	PLWH, Male: 76, Female: 26	102	NR	Sex, socioprofessional status, CMV, work status, mode of contamination	118	NR
Liu et al. [37]	MOS-HIV *Chinese version*	China	Chinese	Cross-sectional study	PLWH, age (years) < 20: 15, 20–30: 195 30–40: 158, > 40: 267 Male: 447, Female: 188	635	Between May 2015 and March 2016	General health, physical function, role function, cognitive function, pain, mental health, energy/fatigue, health distress, social function, quality of life, health transition	35	44.1–85.2
Mast et al. [41]	MOS-HIV *Lugandan version*	Uganda	Ugandan	Cross-sectional study	HIV-positive women	803	NR	Perceived health, bodily pain, QoL, role functioning, social functioning, vitality, mental health, health distress, cognitive functioning, physical functioning, health transition	35	0–100
McDonne et al. [42]	MOS-HIV	US	English	Cross-sectional study	HIV-positive nonpregnant women, mean age: 33	287	Between April 1993 and June 1995	Cognitive functioning, physical functioning, social functioning, role functioning, mental health, health distress, overall QoL	17	0–100
Meemon et al. [50]	WHOQOL-HIV-BREF *Thai version*	Thailand	Thai	Cross-sectional study	PLWH, mean age: 41.95 ± 7.82 Male: 146, Female: 183	329	Between August and October 2014	Physical health; psychological health; level of independence; social relationships; environmental health; spirituality, religion, and personal beliefs	31	4–20
Namisango et al. [24]	MVQoLI *Uganda version*	Uganda	Ugandan	Cross-sectional study	Advanced AIDS Age (years) 18–29: 39, 30–39: 97, 40 + : 64, Male: 78, Female: 122	200	NR	Symptoms, functional status, interpersonal relations, emotional well-being, transcendence	25	NR
Nosyk et al. [28]	HUI3	Canada	English	Cross-sectional study	Patients with advanced HIV/AIDS Mean age: 48 years (SD: 8.5) Male: 361, Female: 7	368	Between June 2006 and December 2007			NR
Patel et al. [18]	SF-12 *Kiswahili version*	Kenya	Kiswahili	Cross-sectional study	Kiswahili-speaking PLWH Male: 76, Female: 26	102	Between May 2007 and October 2009		12	0.35–1
Paton et al. [43]	MOS-HIV *English and Chinese versions*	Singapore	English, Chinese	Cross-sectional study	HIV-infected patients Mean age: 38 Male: 156, Female: 7	163	Between April and August 1998	Overall health, pain, physical functioning, role functioning, social functioning, mental health, energy/fatigue, health distress, cognitive functioning, quality of life, health transition	30	0–100
Pereira et al. [52]	WHOQOL-HIV-Bref *Portuguese version*	Portugal	Portuguese	Cross-sectional study	HIV-infected patients aged 50 years and older Mean age: 57.84 (6.79,50–81) Male: 120, Female: 65	185	NR	Six domains (physical, psychological, independence, social relationships, environment, spirituality) and 29 specific facets	31	NR
Pereira and Canavarro [75]	EUROHIS-QoL-8 *Portuguese version*	Portugal	Portuguese	Cross-sectional study	PLWH, mean age: 40.72 (SD = 9.71, range: 18–81) Male: 808, Female: 389	1197	Between September 2007 and July 2008	Overall QoL, general health, energy, daily activities, self-esteem, relationships, financial resources, living place	8	NR
Pereira and Canavarro [75]	BSI *Portuguese version*	Portugal	Portuguese	Cross-sectional study	PLWH, mean age 40.72 (SD = 9.71, range: 18–81) Male: 808, Female: 389		NR	NR	53	0–4
Peterman et al. [15]	FAHI	US	English	Cross-sectional study	PLWH, Male: 307, Female: 54	361	NR	Physical well-being, function and global well-being, emotional well-being/living with HIV, social well-being, cognitive functioning	44	0–176
Remple et al. [66]	MQoL-HIV	Canada	English	Cross-sectional study	HIV-infected Women Mean age: 36.5 years (SD = 9.5)	85	NR	Mental health, physical functioning, physical health, social support, social functioning, cognitive functioning, financial status, partner intimacy, sexual functioning, medical care	40	4–28
Reychler et al. [48]	WHOQOL-HIV *French version*	France, the francophone part of Belgium	French	Cross-sectional study	PLWH Male: 32 (64.0) Female: 18 (36.0)	50	NR	Six domains (physical, psychological, level of dependence, social relationships, environment and spirituality) and 29 facets	120 items and 37 important questions	NR
Riley et al. [72]	SF-36	US	English	Cross-sectional study	PLWH, mean age: 39 Male: 274, Female: 56	330	NR	General health perceptions, physical functioning, role limitations due to physical problems, role limitations due to emotional problems, social functioning, bodily pain, vitality, mental health	36	NR
Saddkia et al. [9]	WHOQOL *Malay version*	Malaysia	Malay	Cross-sectional study	PLWH, mean age (years): 35.7 (7.50) Male: 94 (59.9) Female: 63 (40.1)	157	Between August and December 2007	Physical, psychological, level of independence, social relationships, environment, spirituality	31	4–20
Salehi et al. [53]	WHOQOL-HIV BREF *Persian version*	Islamic Republic of Iran	Persian	Cross-sectional study	PLWH, mean age: 38.06 (9.32) Male: 44, Female: 17	61	NR	Physical, psychological, level of independence, social relationship, environmental, spiritual	29	1.6–6.6
Schifano et al. [44]	MOS-HIV *Italian version*	Italy	Italian	Cross-sectional study	PLWH Males: 135, Females: 50 Age (years) 21–30: 35, 31–35: 65, > 35: 85	185	Between October 1994 and April 1996	Physical functioning, social functioning, role functioning, bodily pain, mental health, health distress, cognitive functioning, vitality, general health, health perception	35	0–100
Schnall et al. [25]	PROMIS-29	US	English	Cross-sectional study	PLWH, mean age (years) (SD): 48.5 (11.70) Male: 933, Female: 359 Transgender male/transman/FTM: 2 Transgender female/transwoman/MTF: 8 Genderqueer individual: 4	1306	Between February and July 2016	Physical functioning, anxiety, depression, fatigue, sleep disturbance, satisfaction with participation in social roles, pain interference and pain intensity	29	1–5
Shim et al. [45]	MOS-HIV *Korean version*	South Korea	Korean	Cross-sectional study	PLWH, age ≤ 40: 54, 41–60: 107, > 60: 40, Male: 179, Female: 22	201	Between December 2016 and June 2017	General health perception, pain, physical functioning, role functioning, social functioning, energy/fatigue, mental health, health distress, cognitive functioning, quality of life, health transition	35	0–100
Smith et al. [65]	MOS SF-20	US	English	Cross-sectional study	Women with HIV Mean age: 33.5 (± 7.69)	202	NR	Physical functioning, role functioning, social functioning, mental health, general health perceptions, pain	20	0–100
Smith et al. [67]	MQoL-HIV	US	English	Cross-sectional study	PLWH Male: 95, Female: 26	121	Between July 1994 and December 1995	Mental health, physical health, physical functioning, social functioning, social support, cognitive functioning, financial status, partner intimacy, sexual functioning, medical care	40	NR
Sousa et al. [26]	HAQ-DI	US	English	Cross-sectional study	PLWH, mean age: (39.35 ± 8.13) (61.57 ± 12.46) Male: 917, Female: 901	1818	NR	Usual activities, reaching, grip, eating, dressing/grooming, hygiene, walking, arising	20	0–3
Stangl et al. [46]	MOS-HIV *Ugandan version*	Uganda	Ugandan	Cross-sectional study	PLWH, Male: 237, Female: 710 Age 18–30: 159 31–40: 434, 41 + : 354	947	Between May 2003 and May 2004	Physical function, role function, general health perceptions, bodily pain, health transition, mental health, cognitive function, health distress, social function, vitality		NR
Starce et al. [49]	WHOQOL-HIV *Italian version*	Italy	Italian	Cross-sectional study	PLWH Male: 105, Female: 46	151	NR	Physical, psychological, level of independence, social relationships, environment, spirituality, religion, personal beliefs of PLWH	28	0–100
Stasinopouiou et al. [34]	MOS-HIV *Greek version*	Greece	Greek	Cross-sectional study	PLWH, mean age (SD): 42.6 (9.4) Male: 118, Female: 36	154	NR	Quality of life, pain, physical functioning, role functioning, social functioning, mental health, energy/fatigue, cognitive function, health distress, health transition	35	0–100
Taylor et al. [47]	HAT-QoL *Shona version*	Zimbabwe	Shona	Cross-sectional study	Patients with HIV-related opportunistic infections Teens: 32, 20–29: 164 30–39: 136, 40–49: 52 50–59: 20, 60–69: 4 Female: 232, Male: 168	400	NR	Overall function (physical, role, and social function), sexual function, disclosure worries, health worries, financial worries, HIV mastery, life satisfaction, medication worries, provider trust	34	0–100
Taylor et al. [47]	MOS-HIV-35 *Shona version*	Zimbabwe	Shona	Cross-sectional study	Patients with HIV-related opportunistic infections Teens: 32, 20–29: 164 30–39: 136, 40–49: 52 50–59: 20, 60–69: 4 Female: 232, Male: 168	400	NR	General health perceptions, physical function, role function, social function, cognitive function, pain, mental health, energy/fatigue, health distress, overall QoL	35	NR
Tesfaye et al. [54]	WHOQOL-HIV-BREF *Ethiopian version*	Ethiopia	Ethiopian	Cross-sectional study	PLWH Mean age: 32.5 (7.9) Male: 38, Female: 62	100	NR	Physical, psychological, independence, social relationships, environment, spirituality	27	NR
Thompson et al. [64]	WHOQOL-BREF	US	English	Cross-sectional study	PLWH	312	NR	Physical health, psychological health, social relationships, environmental conditions	24	26–130
Tran [55]	WHOQOL-HIV-BREF *Vietnamese version*	Vietnam	Vietnamese	Cross-sectional study	PLWH, age ≤ 35 years old: 584 > 35 years old: 432 Male: 648, Female: 368	1016	NR	Physical, morbidity, social, spirituality, performance, environment	31	4–20
Tran et al. [20]	EQ-5D-5L *Vietnamese version*	Vietnam	Vietnamese	Cross-sectional study	PLWH, age ≤ 35 years old: 584 > 35 years old: 432 Male: 648, Female: 368	1016	NR	Mobility, self-care, usual activities, pain/discomfort and anxiety/depression	25	NR
Turner-Bowker et al. [73]	SF-36	US	English	Cross-sectional study	PLWH Male: 117, Female: 84	201	NR	Physical function, role function (without physical or emotional attribution), bodily pain, general health, vitality, social function, mental health	36	NR
Watanabe et al. [68]	MQoL-HIV *Japanese version*	Japan	Japanese	Cross-sectional study	PLWH, mean age (years) 36.5 + 10.3 Male: 344, Female: 31	375	Between January and May 2000	Mental health, physical health, physical functioning, social functioning, social support, cognitive functioning, financial status, partner intimacy, sexual functioning, medical service	40	12–84
WHOQOL-HIV Group [13]	WHOQOL-HIV *Australian, Indic, Brazilian, Thai, and Zimbabwean versions*	Australia, India, Brazil, Thailand, Zimbabwe	Australian, Indic, Brazilian, Thai, Zimbabwean	Cross-sectional study	PLWH, mean age 32.3 (79.4) Male: 569, Female: 331	900	NR	Physical, psychological, independence, social, environmental and spirituality	25	4–20
Wu et al. [21]	EQ-5D	US	English	Cross-sectional study	PLWH Male: 931, Female: 59 Mean age: 38.5 (SD: 7.8)	990	NR	Anxiety/depression, mobility, usual activities, pain/discomfort and self-care		0–100
Wu et al. [21]	MOS-HIV	US	English	Cross-sectional study	PLWH Male: 931, Female: 59 Mean age: 38.5 (SD: 7.8)	990	NR	General health perceptions, cognitive functioning, pain, physical functioning, role functioning, health distress, quality of life, mental health and energy/fatigue	35	0–100
Zhu et al. [58]	WHOQOL HLV BREF *Chinese version*	China	Chinese	Cross-sectional study	PLWH, mean age: 39.62 (12.73) Male: 965, Female: 135	1100	NR	General QoL, general health status, physical, psychological, independence, social relationships, environment, spirituality	31	4–20
Cai et al. [59]	WHOQOL HLV BREF *Chinese version*	China	Chinese	Cross-sectional study	PLWH, mean age: 36.8 Male: 105, Female: 33	138	NR	Physical, psychological, level of independence, social relationship, environmental, spiritual	31	4–20
Chen et al. [60]	WHOQOL HLV BREF *Chinese version*	China	Chinese	Cross-sectional study	PLWH, mean age: 38.29 ± 10.92 Male: 72, Female: 30	102	NR	Physical, psychological, level of independence, social relationship, environmental, spiritual	31	NR
Dong et al. [38]	MOS-HIV *Chinese version*	China	Chinese	Cross-sectional study	PLWH, Male: 185, Female: 44	229	Between April 2012 and April 2013	Physical function, role function, general health perceptions, bodily pain, health transition, mental health, cognitive function, health distress, social function, vitality	35	0–100
Guo et al. [82]	HIV QoL Scale-4	China	Chinese	Cross-sectional study	PLWH, mean age: 42.67 ± 7.67 Male: 40, Female: 68	108	NR	Physical function, psychological function, social function, general health	49	1–5
Liu et al. [62]	WHOQOL-HIV *Chinese version*	China	Chinese	Cross-sectional study	PLWH, mean age: 43.83 ( ±) 7.44 Male: 32, Female: 56	88	NR	Physical, psychological, level of independence, social relationship, environmental, spiritual	31	NR
Luo et al. [61]	WHOQOL-HIV-BREF *Chinese version*	China	Chinese	Cross-sectional study	PLWH, mean age: 18 ~ 78 (38.29 ± 12.90) Male: 93 Female: 31	124	Between September 2012 and June 2013	Physical, psychological, level of independence, social relationship, environmental	31	NR
Meng et al. [80]	HIV QoL Scale-2	China	Chinese	Cross-sectional study	PLWH, mean age: 35.9 Male: 292, Female: 151	443	Between July 2005 and October 2006	Mental status, concerns of health and responsibility, family social support, hostile psychological trends, vitality, appetite and pain, economic concerns, doctor support, alienation, life satisfaction	44	0–100
Su et al. [81]	HIV QoL Scale-3	China	Chinese	Cross-sectional study	PLWH, mean age: 42.67 ± 7.67 Male: 40, Female: 68	108	Between October 2004 and December 2006	Physical function, psychological function, social function, general health	49	1–5
Xiang et al. [77–79]	HIV QoL Scale-1	China	Chinese	Cross-sectional study	PLWH, mean age: 40 ± 9 Male: 195, Female: 162	353	NR	Physical, psychological, social	55	NR
Yang et al. [39]	MOS -HIV *Chinese version*	China	Chinese	Cross-sectional study	PLWH, mean age 35.2 Male: 80, Female: 37	117	NR	Physical function, role function, general health perceptions, bodily pain, health transition, mental health, cognitive function, health distress, social function, vitality	35	NR
Yu et al. [40]	MOS-HIV *Chinese version*	China	Chinese	Cross-sectional study	PLWH, mean age: 40.77 ± 8.81 Male: 422, Female: 336	758	NR	Physical function, role function, general health perceptions, bodily pain, health transition, mental health, cognitive function, health distress, social function, vitality	35	NR
Zhang et al. [17]	HIV/AIDSQoL-46 *Chinese version*	China	Chinese	Cross-sectional study	PLWH	240	NR	Physical function, psychological function, social function, general feeling	46	NR
Zhang et al. [74]	SF-36 *Chinese version*	China	Chinese	Cross-sectional study	PLWH, age < 35: 98, ≥ 35: 141, ≥ 45: 55 Male: 227, Female: 67	294	NR	Physical function, role function, bodily pain, general health, vitality, social function, mental health	35	NR

EQ-5D, EuroQol-5 Dimensions; EUROHIS-QoL-8, European health interview surveys-quality of life-8; FAHI, Functional Assessment of HIV Infection; HAT-QoL, HIV/AIDS Targeted Quality of Life; HAQ-DI, Health Assessment Questionnaire Disability Index; HIV-QL31, HIV Disease Quality of Life 31-Item Instrument; HIV/AIDSQoL, HIV/AIDS Quality of Life Questionnaire; HOPES, HIV Overview of Problems Evaluation Scale; HUI3, Health Utility Index 3; ISSQoL, The Italian National Institute of Health Quality of Life; MQoL-HIV, Multidimensional Quality of Life Questionnaire for Persons with HIV/AIDS; MOS, Medical Outcomes Study; MOS-HIV, Medical Outcomes Study HIV Health Survey; MVQoLI, Missoula-Vitas Quality-of-Life Index; NR, not reported; PLWH, people living with HIV; PROM, Patient-reported outcome measure; PROMIS, Patient-Reported Outcomes Measurement Information System; PROQoL-HIV, Patient-Reported Outcomes Quality of Life-HIV instrument; QWB, Quality of Well-Being scale; SF, Short Form Health Survey; WHOQoL, World Health Organization's Quality of Life; WHOQoL-BREF, The brief version of the World Health Organization's Quality of Life. WHOQoL-HIV, World Health Organization's Quality of Life Instrument in HIV Infection; WHOQoL-HIV-BREF, The brief version of the World Health Organization's Quality of Life Instrument in HIV Infection

The characteristics of all 30 HRQoL PROMs, including the items, domains, and score range, are shown in Table 1. The total number of items ranged from 8 to 142 [9–28, 34–82]. A total of 10 PROMs had multiple language versions, and the remaining 18 had only one language version. Tables 4 and 5 summarize the psychometric properties of the HIV-specific and generic instruments.

### Quality assessment

#### Methodological quality assessment

Tables 2 and 3 show the methodological quality of the 69 included studies based on the COSMIN checklist. All studies were considered to have sufficient methodological quality for further study. Table 2 presents an overview of the COSMIN ratings of the HIV-specific instruments, and Table 3 presents the generic instruments. Limited information was retrieved on cross cultural validity/translation (58 studies) [11–14, 16–23, 25–28, 35–40, 42–44, 47, 48, 50–56, 58–64, 66–82], criterion validity (59 studies) [9–12, 15–17, 19–26, 34, 37–50, 52–67, 69–79, 81, 82], reliability (49 studies) [11, 13–21, 23–28, 34–36, 38, 39, 41–47, 49–55, 57, 59, 62–65, 68, 69, 72–76], hypothesis testing (18 studies) [11, 16, 17, 34, 38, 39, 41, 53, 61, 67, 68, 71, 77–82] and responsiveness (62 studies) [9–16, 18–20, 22–27, 34–45, 47–57, 59–64, 66, 68–82]. No data were identified on error and interpretability.

**Table 2:** Methodological quality assessment of the HIV-specific instruments

References	PROM	Measurement property: methodological quality per study										
		PROM development	Relevance	Comprehensiveness	Comprehensibility	Construct validity	Internal consistency	Cross‐cultural validity\measurement invariance	Criterion validity	Reliability	Hypothesis testing for construct validity	Responsiveness
Ahmed e t al [56]	WHOQOL-HIV-BREF *Urdu version*	Inadequate	NR	NR	NR	Inadequate	Very good	NR	NR	Doubtful	Very good	NR
Connell and Skevington [51]	WHOQOL-HIV-BREF	Inadequate	NR	NR	NR	Very good	Very good	NR	Very good	NR	Very good	NR
De Boer et al. [14]	HOPES *Dutch and English versions*	Inadequate	NR	NR	NR	Inadequate	Very good	NR	NR	NR	Very good	NR
Duracinsky et al. [12]	PROQoL-HIV *English, Brazilian, Cambodia, Chinese, French, Senegalese, and Thai versions*	Inadequate	NR	NR	NR	Adequate	Very good	NR	Very good	Doubtful	Very good	NR
Herrmann et al. [71]	PROQoL-HIV	Inadequate	NR	NR	NR	NR	Very good	NR	NR	Doubtful	NR	NR
Holmes and Shea [16]	HAT-QoL	Inadequate	NR	NR	NR	Adequate	Very good	NR	NR	NR	NR	NR
Holmes and Shea [76]	HAT-QoL	Inadequate	NR	NR	NR	Very good	Very good	NR	NR	NR	Very good	NR
Hsiung et al. [57]	WHOQOL-HIV-BREF *Chinese version*	Inadequate	Doubtful	Doubtful	Doubtful	Very good	Very good	Very good	NR	NR	Very good	NR
Hughes et al. [35]	MOS-HIV	Inadequate	NR	NR	NR	Inadequate	Very good	NR	NR	NR	Very good	NR
Kemmler et al. [10]	MQoL-HIV *German version*	Inadequate	Doubtful	Doubtful	Doubtful	Adequate	Very good	Doubtful	Inadequate	Very good	Very good	NR
Lau et al. [36]	MOS-HIV *Chinese version*	Inadequate	NR	NR	NR	Adequate	Very good	NR	Very good	NR	Very good	NR
Leplège et al. [11]	HIV-QL31 *French version*	Inadequate	Doubtful	Doubtful	Doubtful	Inadequate	Very good	NR	NR	NR	NR	NR
Liu et al. [37]	MOS-HIV *Chinese version*	Inadequate	NR	NR	NR	Very good	Very good	NR	NR	Doubtful	Very good	NR
Mast et al. [41]	MOS-HIV *Ugandan version*	Inadequate	NR	NR	NR	Adequate	Very good	Very good	NR	NR	NR	NR
McDonneet al. [42]	MOS-HIV	Inadequate	NR	NR	NR	Adequate	Very good	NR	NR	NR	Very good	NR
Meemon et al. [50]	WHOQOL-HIV-BREF *Thai version*	Inadequate	NR	NR	NR	Very good	Very good	NR	NR	NR	Very good	NR
Paton et al. [43]	MOS-HIV	Inadequate	NR	NR	NR	NR	Very good	NR	NR	NR	Very good	NR
Pereira et al. [52]	WHOQOL-HIV-Bref *Portuguese version*	Inadequate	NR	NR	NR	Adequate	Very good	NR	NR	NR	Very good	NR
Peterman et al. [15]	FAHI	Inadequate	NR	NR	NR	Adequate	Inadequate	Doubtful	NR	NR	Very good	NR
Remple et al. [66]	MQoL-HIV	Inadequate	Doubtful	Doubtful	Doubtful	NR	Very good	NR	NR	Doubtful	Very good	NR
Reychler et al. [48]	WHOQOL-HIV *French version*	Inadequate	NR	NR	NR	Inadequate	Inadequate	NR	NR	Doubtful	Very good	NR
Salehi et al. [53]	WHOQOL-HIV-BREF *Persian version*	Inadequate	Doubtful	Doubtful	Doubtful	Inadequate	Very good	NR	NR	NR	NR	NR
Schifano et al. [44]	MOS-HIV *Italian version*	Inadequate	NR	NR	NR	Adequate	Very good	NR	NR	NR	Very good	NR
Shim et al. [45]	MOS-HIV *Korean version*	Inadequate	NR	NR	NR	Adequate	Very good	Very good	NR	NR	Very good	NR
Smith et al. [67]	MQoL-HIV	Inadequate	Doubtful	Doubtful	Doubtful	Adequate	Very good	Adequate	NR	Adequate	NR	NR
Stangl et al. [46]	MOS-HIV *Ugandan version*	Inadequate	Doubtful	Doubtful	Doubtful	Adequate	Very good	Very good	NR	NR	Inadequate	NR
Starce et al. [49]	WHOQOL-HIV *Italian version*	Inadequate	Doubtful	Doubtful	Doubtful	Inadequate	Very good	Inadequate	NR	NR	NR	NR
Stasinopouiou et al. [34]	MOS-HIV *Greek version*	Inadequate	NR	NR	NR	Adequate	Very good	Very good	NR	NR	NR	NR
Taylor et al. [47]	HAT-QoL *Shona version*	Inadequate	Doubtful	Doubtful	Doubtful	Inadequate	Very good	Inadequate	NR	NR	Inadequate	NR
Taylor et al. [47]	MOS-HIV *Shona version*	Inadequate	Doubtful	Doubtful	Doubtful	Inadequate	Very good	Inadequate	NR	NR	Inadequate	NR
Tesfaye et al. [54]	WHOQOL-HIV-BREF *Ethiopian version*	Inadequate	NR	NR	NR	Very good	Very good	NR	NR	NR	Very good	NR
Tran [55]	WHOQOL-HIV-BREF *Vietnamese version*	Inadequate	NR	NR	NR	Adequate	Inadequate	NR	NR	NR	Very good	NR
Watanabe et al. [68]	MQoL-HIV *Japanese version*	Inadequate	NR	NR	NR	Adequate	Very good	NR	NR	NR	NR	NR
WHOQOL-HIV Group [13]	WHOQOL-HIV	Inadequate	NR	NR	NR	NR	Very good	NR	Very good	NR	Very good	NR
Wu et al. [21]	MOS-HIV	Inadequate	NR	NR	NR	Inadequate	Inadequate	NR	NR	NR	Very good	NR
Zhu et al. [58]	WHOQOL HLV BREF *Chinese version*	Inadequate	NR	NR	NR	Very good	Very good	NR	NR	Very good	Very good	NR
Cai et al. [59]	WHOQOL HLV BREF *Chinese version*	Inadequate	Doubtful	Doubtful	Doubtful	Adequate	Inadequate	NR	NR	NR	Very good	NR
Chen et al. [60]	WHOQOL HLV BREF *Chinese version*	Inadequate	NR	NR	NR	Inadequate	Inadequate	NR	NR	Inadequate	NR	NR
Dong et al. [38]	MOS—HIV *Chinese version*	Inadequate	NR	NR	NR	Very good	Inadequate	NR	NR	NR	NR	NR
Guo et al. [82]	HIV QoL Scale-4	Inadequate	NR	NR	NR	Very good	Inadequate	NR	NR	Very good	NR	NR
Liu et al. [62]	WHOQOL *Chinese version*	Inadequate	NR	NR	NR	Doubtful	Inadequate	NR	NR	NR	NR	NR
Luo et al. [61]	WHOQOL-HIV-BREF *Chinese version*	Inadequate	NR	NR	NR	Adequate	Inadequate	NR	NR	Very good	NR	NR
Meng et al. [80]	HIV QoL Scale-2	Inadequate	NR	NR	NR	Very good	Inadequate	NR	Very good	Very good	Very good	NR
Su et al. [81]	HIV QoL Scale-3	Inadequate	Doubtful	Doubtful	Doubtful	Very good	Inadequate	NR	NR	Very good	NR	NR
Xiang et al. [77–79]	HIV QoL Scale-1	Inadequate	Doubtful	Doubtful	Doubtful	Adequate	Inadequate	Doubtful	Very good	Very good	Very good	NR
Yang et al. [39]	MOS –HIV *Chinese version*	Inadequate	Doubtful	Doubtful	Doubtful	Adequate	Inadequate	NR	NR	NR	NR	NR
Yu et al. [40]	MOS-HIV *Chinese version*	Inadequate	NR	NR	NR	Adequate	Inadequate	NR	NR	Very good	NR	NR
Zhang et al. [17]	HIV/AIDSQoL-46 *Chinese version*	Inadequate	NR	NR	NR	Very good	Inadequate	NR	NR	Very good	NR	Very good

FAHI, Functional Assessment of HIV Infection; HAT-QoL, HIV/AIDS Targeted Quality of Life; HIV-QL31, HIV Disease Quality of Life 31-Item Instrument; HIV/AIDSQoL, HIV/AIDS Quality of Life Questionnaire; HOPES, HIV Overview of Problems Evaluation Scale; MQoL-HIV, Multidimensional Quality of Life Questionnaire for Persons with HIV/AIDS; MOS-HIV, Medical Outcomes Study HIV Health Survey; NR, not reported; PROM, Patient-reported outcome measure; PROQoL-HIV, Patient-Reported Outcomes Quality of Life-HIV instrument; WHOQoL-HIV, World Health Organization's Quality of Life Instrument in HIV Infection; WHOQoL-HIV-BREF, The brief version of the World Health Organization's Quality of Life Instrument in HIV Infection

**Table 3:** Methodological quality assessment of the generic instruments

References	PROM	Measurement property: methodological quality per study										
		PROM development	Relevance	Comprehensiveness	Comprehensibility	Construct validity	Internal consistency	Cross‐cultural validity\measurement invariance	Criterion validity	Reliability	Hypothesis testing for construct validity	Responsiveness
Akinboro et al. [63]	WHOQOL-BREF *Nigerian version*	Inadequate	NR	NR	NR	NR	Very good	NR	NR	NR	NR	NR
Brown et al. [70]	PozQoL	Inadequate	NR	NR	NR	Very good	Very good	NR	NR	Doubtful	Very good	NR
Bucciardini et al. [69]	ISSQoL *Italian version*	Inadequate	NR	NR	NR	Inadequate	Very good	NR	NR	NR	NR	NR
Fang et al. [22]	WHOQOL *Chinese version*	Inadequate	NR	NR	NR	Adequate	Very good	NR	NR	Doubtful	Very good	NR
Kaplan et al. [27]	QWB scale	Inadequate	NR	NR	NR	NR	NR	NR	Very good	NR	NR	NR
Kohli et al. [23]	MOS *Indic version*	Inadequate	NR	NR	NR	Inadequate	Very good	NR	NR	NR	NR	NR
Kusterer et al. [19]	SF-36v2 *Brazilian-Portuguese version*	Inadequate	NR	NR	NR	Very good	Very good	NR	NR	NR	NR	NR
Namisango et al. [24]	MVQoLI *Uganda version*	Inadequate	Doubtful	Doubtful	Doubtful	Very good	Very good	Very good	Very good	Doubtful	Very good	NR
Nosyk et al. [28]	HUI3	Inadequate	NR	NR	NR	NR	NR	NR	Adequate	NR	Very good	NR
Patel et al. [18]	SF-12 *Kiswahili version*	Inadequate	Doubtful	Doubtful	Doubtful	Very good	NR	NR	Very good	NR	NR	NR
Pereira and Canavarro [75]	EUROHIS-QoL-8 *Portuguese version*	Inadequate	NR	NR	NR	Very good	NR	NR	NR	NR	Very good	NR
Riley et al. [72]	SF-36	Inadequate	NR	NR	NR	Very good	Very good	NR	NR	NR	Very good	NR
Saddkia et al. [9]	WHOQOL *Malay version*	Inadequate	NR	NR	NR	Adequate	Very good	Adequate	NR	Doubtful	Very good	NR
Schnall et al. [25]	PROMIS-29	Inadequate	NR	NR	NR	Adequate	Very good	NR	NR	Doubtful	NR	NR
Smith et al. [65]	MOS SF-20	Inadequate	Doubtful	Doubtful	Doubtful	Inadequate	Inadequate	Adequate	Very good	Doubtful	Very good	NR
Sousa et al. [26]	HAQ-DI	Inadequate	Doubtful	Doubtful	Doubtful	Adequate	Inadequate	Very good	Very good	Doubtful	Very good	NR
Thompson et al. [64]	WHOQOL-BREF	Inadequate	NR	NR	NR	Very good	Inadequate	NR	NR	NR	Very good	NR
Tran et al. [20]	EQ-5D-5L *Vietnamese version*	Inadequate	NR	NR	NR	Inadequate	Inadequate	Very good	Very good	NR	Very good	NR
Turner-Bowker et al. [73]	SF-36	Inadequate	NR	NR	NR	Inadequate	NR	NR	NR	NR	Very good	NR
Wu et al. [21]	EQ-5D	Inadequate	NR	NR	NR	Inadequate	Inadequate	NR	Very good	NR	Very good	NR
Liu et al. [62]	WHOQOL *Chinese version*	Inadequate	NR	NR	NR	Doubtful	Inadequate	NR	NR	NR	NR	NR
Zhang et al. [74]	SF-36 *Chinese version*	Inadequate	NR	NR	NR	Very good	Inadequate	NR	Very good	NR	Very good	NR

EQ-5D, EuroQol-5 Dimensions; EUROHIS-QoL-8, European health interview surveys-quality of life-8; HAQ-DI, Health Assessment Questionnaire Disability Index; HUI3, Health Utility Index 3; ISSQoL, The Italian National Institute of Health Quality of Life; MOS, Medical Outcomes Study; MVQoLI, Missoula-Vitas Quality-of-Life Index; NR, Not reported; PROM, Patient-reported outcome measure; PROMIS, Patient-Reported Outcomes Measurement Information System; QWB, Quality of Well-Being scale; SF, Short Form Health Survey; WHOQoL, World Health Organization's Quality of Life; WHOQoL-BREF, The brief version of the World Health Organization's Quality of Life

#### Quality of measurement properties of assessments

Table 4 presents the quality of the psychometric properties retrieved from the 69 included studies for all 30 measures. Fifteen PROMs were rated as insufficient (-) for content validity [11, 17, 48, 49, 53, 57, 59–61, 77–82]. There were 19 PROMs [19, 24, 26, 37–40, 45, 51–54, 57, 59, 60, 64, 70, 74, 75] rated as sufficient (+) for construct validity, and 31 [10–12, 14–17, 21, 34–36, 41, 42, 44, 46–48, 50, 53, 55, 56, 58, 59, 67, 68, 76–79, 81, 82] were rated as insufficient (−). The internal consistency was rated as sufficient (+) for 59 PROMs [9–19, 22–25, 34, 36–49, 51–72, 74, 76–82] and as insufficient (−) for 4 PROMs [20, 21, 35, 50].

**Table 4:** Rating of the measurement properties of the instruments

PROM	References	Construct validity (CFI)	Internal consistency (Cronbach’s alpha)	Reliability (ICC)	Measurement error	Hypothesis testing for construct validity	Cross‐cultural validity\measurement invariance	Criterion validity	Responsiveness
WHOQOL-HIV	WHOQOL-HIV Group [13]	NR	+ (0.87–0.94)	NR	NR	?	NR	−	NR
WHOQOL-HIV *French version*	Reychler et al. [48]	−	+ (0.94)	−(0.42–0.74)	NR	?	NR	NR	NR
WHOQOL-HIV *Italian version*	Starce et al. [49]	NR	+ (0.53–0.89)	NR	NR	?	+	NR	NR
WHOQOL-HIV-BREF	Connell and Skevington [51]	+ (0.97)	+ (0.74–0.82)	NR	NR	+	NR	−	NR
WHOQOL-HIV-BREF *Thai version*	Meemon et al. [50]	−	+ (0.91)	NR	NR	+	NR	NR	NR
WHOQOL-HIV-BREF *Portuguese version*	Pereira et al. [52]	+ (0.97)	+ (0.65–0.86)	NR	NR	+	NR	NR	NR
WHOQOL-HIV BREF *Persian version*	Salehi et al. [53]	−	+ (0.87)	NR	NR	NR	NR	NR	NR
WHOQOL-HIV-BREF *Ethiopian version*	Tesfaye et al. [54]	+ (0.82)	+ (0.93)	NR	NR	+	NR	NR	NR
WHOQOL-HIV-BREF *Vietnamese version*	Tran [55]	−	+ (0.67–0.89)	NR	NR	?	NR	NR	NR
WHOQOL-HIV-BREF *Urdu version*	Ahmed et al. [56]	−	+ (0.93)	+ (0.87–0.99)	NR	?	NR	NR	NR
WHOQOL-HIV-BREF *Chinese version*	Hsiung et al. [57]	+ (0.95)	+ (0.67–0.80)	NR	NR	+	+	NR	NR
	Zhu et al. [58]	−(0.81)	+ (0.93)	+ (0.72–0.82)	NR	+	NR	NR	+
	Cai et al. [59]	−	+ (0.60–0.82)	NR	NR	+	NR	NR	NR
	Chen et al. [60]	−	+ (> 0.60)	−(> 0.50)	NR	+	NR	NR	NR
	Luo et al. [61]	−	+ (0.60–0.76)	+ (0.47–0.68)	NR	NR	NR	NR	NR
MOS-HIV	Hughes et al. [35]	−	−(0.57–0.89)	NR	NR	+	NR	−	NR
	McDone et al. [42]	−	+ (0.64–0.89)	NR	NR	?	NR	NR	NR
	Paton et al. [43]	NR	+ (> 0.70)	NR	NR	+	NR	NR	NR
	Wu et al. [21]	−	−	NR	NR	?	NR	NR	+
MOS-HIV *Greek version*	Stasinopo et al. [34]	−	+ (> 0.80)	NR	NR	NR	+	NR	NR
MOS-HIV *Chinese version*	Lau et al. [36]	-	+ (0.78–0.90)	+ (0.50–0.84)	NR	NR	NR	NR	NR
	Liu et al. [37]	+ (0.97)	+ (0.79–0.93)	+ (0.87–0.89)	NR	+	NR	NR	NR
	Dong et al. [38]	−	+ (0.81)	NR	NR	NR	NR	NR	NR
	Yang et al. [39]	−	+ (0.67–0.86)	NR	NR	NR	NR	NR	NR
	Yu et al. [40]	−	+ (0.69–0.87)	+ (0.73–0.88)	NR	+	NR	NR	NR
MOS-HIV *Lugandan version*	Mast et al. [41]	−	+ (0.51–0.84)	NR	NR	NR	+	NR	NR
MOS-HIV *Italian version*	Schifano et al. [44]	−	+ (> 0.80)	NR	NR	?	NR	NR	NR
MOS-HIV *Korean version*	Shim et al. [45]	+ (0.97)	+ (0.78–0.95)	NR	NR	+	+	NR	NR
MOS-HIV *Ugandan version*	Stangl et al. [46]	−	+ (0.79–0.91)	NR	NR	?	+	NR	−
MOS-HIV *Shona version*	Taylor et al. [47]	−	+ (0.60–0.86)	NR	NR	+	NR	NR	NR
MQoL-HIV	Remple et al. [66]	NR	+ (0.43–0.92)	+ (0.60–0.96)	NR	+	NR	NR	NR
	Smith et al. [67]	−	+ (0.56–0.86)	+ (0.64–0.88)	NR	NR	NR	NR	+
MQoL-HIV *German version*	Kemmler et al. [10]	−	+ (0.61–0.85)	+ (0.74–0.89)	NR	+	NR	NR	NR
MQoL-HIV *Japanese version*	Watanabe et al. [68]	−	+ (0.47–0.85)	NR	NR	NR	NR	−	NR
FAHI	Peterman et al. [15]	−	+ (0.91)	NR	NR	?	+	NR	NR
HAT-QoL	Holmes and Shea et al. [76]	−	+ (> 0.80)	NR	NR	?	NR	NR	NR
	Holmes and Shea et al. [16]	−	+ (0.80–0.89)	NR	NR	NR	NR	NR	NR
HAT-QoL *Shona version*	Taylor et al. [47]	−	+ (0.63–0.85)	NR	NR	?	NR	NR	NR
HIV/AIDSQoL *Chinese version*	Zhang et al. [17]	−	+ (0.94)	+ (0.80)	NR	NR	NR	NR	+
HIV-QL31 *French version*	Leplège et al. [11]	−	+ (0.93)	NR	NR	NR	NR	NR	NR
HOPES *Dutch, English version*	De Boer et al. [14]	−	+ (0.80–0.92)	NR	NR	?	NR	−	NR
PROQoL-HIV	Herrmann et al. [71]	NR	+ (0.94)	+ (0.86)	NR	NR	NR	NR	NR
PROQoL-HIV *English, Brazilian, Cambodian, Chinese, French, Senegalese, and Thai versions*	Duracinsky et al. [12]	−	+ (0.77–0.89)	+ (0.86)	NR	?	NR	NR	NR
HIV QoL Scale-1	Xiang et al. [77–79]	−	+ (0.65–0.7)	+ (> 0.7)	NR	NR	NR	NR	NR
HIV QoL Scale-2	Meng et al. [80]	NR	+ (0.90)	+ (0.80)	NR	NR	NR	−	NR
HIV QoL Scale-3	Su et al. [81]	−	+ (0.94)	+ (0.80)	NR	NR	NR	NR	NR
HIV QoL Scale-4	Guo et al. [82]	−	+ (0.94)	+ (0.97)	NR	NR	NR	NR	NR
WHOQOL *Chinese version*	Fang et al. [22]	−	+ (0.74–0.85)	+ (0.51–0.78)	NR	?	NR	NR	NR
	Liu et al. [62]	−	−	NR	NR	NR	NR	NR	NR
WHOQOL *Malay version*	Saddkia et al. [9]	−	+ (0.93)	+ (0.87)	NR	+	+	NR	NR
WHOQOL-BREF *Nigerian version*	Akinboro et al. [63]	NR	+ (0.85)	NR	NR	NR	NR	NR	NR
WHOQOL-BREF	Thompson et al. [64]	+ (0.89)	+ (0.65–0.78)	NR	NR	+	NR	NR	NR
MOS *Indic version*	Kohli et al. [23]	−	+ (> 0.75)	NR	NR	NR	NR	NR	NR
MOS SF-20	Smith et al. [65]	−	+ (0.76–0.89)	NR	NR	NR	+	NR	+
MVQoLI *Uganda version*	Namisango et al. [24]	NR	+ (0.85)	NR	NR	+	+	NR	NR
EQ-5D	Wu et al. [21]	−	−	NR	NR	?	NR	NR	+
EQ-5D-5L *Vietnamese version*	Tran et al. [20]	−	+ (0.85)	NR	NR	?	NR	NR	NR
EUROHIS-QoL-8 *Portuguese version*	Pereira and Canavarro [75]	+ (0.89)	+ (0.85)	NR	NR	+	NR	NR	NR
HAQ-DI	Sousa et al. [26]	+ (0.974)	NR	NR	NR	NR	NR	NR	NR
HUI3	Nosyk et al. [28]	NR	NR	NR	NR	+	NR	−	+
ISSQoL *Italian version*	Bucciardini et al. [69]	−	+ (> 0.70)	NR	NR	NR	NR	NR	NR
PozQoL	Brown et al. [70]	+ (> 0.95)	+ (0.95)	+ (0.95)	NR	+	NR	NR	NR
PROMIS-29	Schnall et al. [25]	−	+ (0.87–0.97)	+ (0.61–0.81)	NR	NR	NR	NR	NR
QWB scale	Kaplan et al. [27]	NR	NR	NR	NR	NR	NR	−	NR
SF-36v2 *Brazilian-Portuguese version*	Kusterer et al. [19]	+ (0.95)	NR	NR	NR	NR	NR	NR	NR
SF-36	Riley et al. [72]	−	+ (0.77–0.90)	NR	NR	?	NR	NR	NR
	Turner-Bowker et al. [73]	NR	NR	NR	NR	?	NR	NR	NR
SF-36 *Chinese version*	Zhang et al. [74]	−	+ (0.928)	NR	NR	+	NR	NR	NR
SF-12 *Kiswahili version*	Patel et al. [18]	−	NR	NR	NR	?	NR	−	NR

“+”, sufficient; “−”, insufficient; “?”, indeterminate; CFI, Comparative fit index; EQ-5D, EuroQol-5 Dimensions; EUROHIS-QoL-8, European health interview surveys-quality of life-8; FAHI, Functional Assessment of HIV Infection; HAT-QoL, HIV/AIDS Targeted Quality of Life; HAQ-DI, Health Assessment Questionnaire Disability Index; HIV-QL31, HIV Disease Quality of Life 31-Item Instrument; HIV/AIDSQoL, HIV/AIDS Quality of Life Questionnaire; HOPES, HIV Overview of Problems Evaluation Scale; HUI3, Health Utility Index 3; ICC, Intra-class correlation coefficients; ISSQoL, The Italian National Institute of Health Quality of Life; MQoL-HIV, Multidimensional Quality of Life Questionnaire for Persons with HIV/AIDS; MOS, Medical Outcomes Study; MOS-HIV, Medical Outcomes Study HIV Health Survey; MVQoLI, Missoula-Vitas Quality-of-Life Index; NR, not reported; PLWH, people living with HIV; PROM,, Patient-reported outcome measure; PROMIS Patient-Reported Outcomes Measurement Information System; PROQoL-HIV, Patient-Reported Outcomes Quality of Life-HIV instrument; QWB, Quality of Well-Being scale; SF, Short Form Health Survey; WHOQoL, World Health Organization's Quality of Life; WHOQoL-BREF, The brief version of the World Health Organization's Quality of Life. WHOQoL-HIV, World Health Organization's Quality of Life Instrument in HIV Infection; WHOQoL-HIV-BREF, The brief version of the World Health Organization's Quality of Life Instrument in HIV Infection

### Certainty of evidence

Table 5 shows the overall quality score for each measurement property of the HIV-specific and generic instruments. Five PROMs were strongly recommended based on the methodological quality of each psychometric property, including MOS-HIV, WHOQoL-HIV-BREF, SF-36, MQoL-HIV, and WHOQoL-HIV. Among the seven language versions of the MOS-HIV [21, 34–47], six were rated as “high” for internal consistency [21, 34, 35, 41–47], and one was rated as “moderate” [36–40]. There were three versions rated as “high” for cross-cultural validity/translation [34, 41, 44, 46]. Among the eight versions of the WHOQoL-HIV-BREF [50–61], five were rated as “high” for internal consistency [50–52, 54, 56], and one was rated as “moderate” [53]. In total, more studies of the MOS-HIV were rated as “high” than studies of the WHOQoL-HIV-BREF, and more studies of the WHOQoL-HIV-BREF were rated as “very low” than studies of the MOS-HIV.

**Table 5:** Overall quality score for each measurement property

Recommendation	PROM	Version	Measurement property: methodological quality per study											
			Relevance	Comprehensiveness	Comprehensibility	Construct validity	Internal consistency	Cross‐cultural validity/measurement invariance	Criterion validity	Reliability	Hypothesis testing for construct validity	Responsiveness	Measurement error	Interpretability
Strongly recommended	MOS-HIV	MOS-HIV	NR	NR	NR	Very low	High	NR	NR	NR	High	NR	NR	NR
		MOS-HIV *Greek version*	NR	NR	NR	Moderate	High	High	NR	NR	NR	NR	NR	NR
		MOS-HIV *Chinese version*	NR	NR	NR	Moderate	Moderate	NR	High	Moderate	High	NR	NR	NR
		MOS-HIV *Ugandan version*	Low	Low	Low	High	High	High	NR	Very low	Moderate	NR	NR	NR
		MOS-HIV *English and Chinese versions*	NR	NR	NR	NR	High	NR	NR	NR	High	NR	NR	NR
		MOS-HIV *Italian version*	NR	NR	NR	Moderate	High	NR	NR	NR	High	NR	NR	NR
		MOS-HIV *Korean version*	NR	NR	NR	Moderate	High	High	NR	NR	High	High	NR	NR
		MOS-HIV *Shona version*	Low	Low	Low	Very low	High	Very low	NR	NR	Very low	NR	NR	NR
	WHOQOL-HIV-BREF	WHOQOL-HIV-BREF	NR	NR	NR	High	High	NR	High	NR	High	NR	NR	NR
		WHOQOL-HIV-BREF *Thai version*	NR	NR	NR	High	High	NR	NR	NR	High	NR	NR	NR
		WHOQOL-HIV-Bref *Portuguese version*	NR	NR	NR	Moderate	High	NR	NR	NR	High	NR	NR	NR
		WHOQOL-HIV-BREF *Persian version*	Very low	Very low	Very low	Very low	Moderate	NR	NR	NR	NR	NR	NR	NR
		WHOQOL-HIV-BREF *Ethiopian version*	NR	NR	NR	High	High	NR	NR	NR	High	NR	NR	NR
		WHOQOL-HIV -BREF *Vietnamese version*	NR	NR	NR	Moderate	Very low	NR	NR	NR	High	NR	NR	NR
		WHOQOL-HIV-BREF *Urdu version*	NR	NR	NR	Very low	High	NR	NR	Low	High	NR	NR	NR
		WHOQOL-HIV-BREF *Chinese version*	High	High	High	Very low	Very low	High	NR	Very low	High	NR	NR	NR
	SF-36	SF-36v2 *Brazilian-Portuguese version*	NR	NR	NR	High	High	NR	NR	NR	NR	NR	NR	NR
		SF-36	NR	NR	NR	Moderate	High	NR	NR	NR	High	NR	NR	NR
		SF-36 *Chinese version*	NR	NR	NR	High	Very low	NR	High	NR	High	NR	NR	NR
	MQoL-HIV	MQoL-HIV	Moderate	Moderate	Moderate	Moderate	High	Moderate	NR	Low	Moderate	High	NR	NR
		MQoL-HIV *German version*	Low	Low	Low	Moderate	High	Low	Very low	High	High	NR	NR	NR
		MQoL-HIV *Japanese version*	NR	NR	NR	Moderate	High	NR	NR	NR	NR	NR	NR	NR
	WHOQOL-HIV	WHOQOL-HIV *English version*	NR	NR	NR	NR	High	NR	High	NR	High	NR	NR	NR
		WHOQOL-HIV *Italian version*	Low	Low	Low	Very low	High	Very low	NR	NR	NR	NR	NR	NR
		WHOQOL-HIV *French version*	NR	NR	NR	Very low	Very low	NR	NR	Very low	Moderate	NR	NR	NR
Weak recommended	FAHI	FAHI	NR	NR	NR	High	Very low	Low	NR	NR	High	NR	NR	NR
	HAT-QoL	HAT-Q oL	NR	NR	NR	Moderate	High	NR	NR	NR	High	NR	NR	NR
		HAT-QoL *Shona version*	Low	Low	Low	Very low	High	Very low	NR	NR	Very low	NR	NR	NR
	HIV/AIDSQoL	HIV/AIDSQoL *Chinese version*	NR	NR	NR	High	Very low	NR	High	NR	NR	NR	NR	NR
	HIV-QL31	HIV-QL31 *French version*	Low	Low	Low	Very low	High	NR	NR	NR	NR	NR	NR	NR
	HOPES	HOPES *Dutch and English versions*	NR	NR	NR	Very low	High	NR	NR	NR	High	NR	NR	NR
	PROQoL-HIV	PROQoL-HIV	NR	NR	NR	Moderate	High	NR	High	Low	High	NR	NR	NR
	WHOQOL	WHOQOL *Chinese version*	NR	NR	NR	Low	Very low	NR	High	Low	High	NR	NR	NR
		WHOQOL *Malay version*	NR	NR	NR	Moderate	High	Moderate	NR	Low	High	NR	NR	NR
	WHOQOL-BREF	WHOQOL-BREF	NR	NR	NR	High	Very low	NR	NR	NR	High	NR	NR	NR
		WHOQOL-BREF *Nigerian version*	NR	NR	NR	NR	High	NR	NR	NR	NR	NR	NR	NR
	MOS	MOS *Indic version*	NR	NR	NR	Very low	Moderate	NR	NR	NR	NR	NR	NR	NR
		MOS SF-20	Low	Low	Low	Very low	Very low	Moderate	High	Low	High	NR	NR	NR
	MVQoLI	MVQoLI *Ugandan version*	Low	Low	Low	High	High	High	High	Low	High	NR	NR	NR
	EQ-5D	EQ-5D	NR	NR	NR	Very low	Very low	NR	High	NR	High	NR	NR	NR
	EQ-5D-5L	EQ-5D-5L *Vietnamese version*	NR	NR	NR	Very low	Very low	High	High	NR	High	NR	NR	NR
	EUROHIS-QoL-8	EUROHIS-QoL-8 *Portuguese version*	NR	NR	NR	High	NR	NR	NR	NR	High	NR	NR	NR
	HAQ-DI	HAQ-DI	Low	Low	Low	Moderate	Very low	High	High	Low	High	NR	NR	NR
	HUI3	HUI3	NR	NR	NR	NR	NR	NR	Moderate	NR	High	NR	NR	NR
	ISSQoL	ISSQoL *Italian version*	NR	NR	NR	Very low	High	NR	NR	NR	NR	NR	NR	NR
	PozQoL	PozQoL	NR	NR	NR	High	High	NR	NR	Low	High	NR	NR	NR
	PROMIS-29	PROMIS-29	NR	NR	NR	Moderate	High	NR	NR	Low	NR	NR	NR	NR
	QWB	QWB	NR	NR	NR	NR	NR	NR	High	NR	NR	NR	NR	NR
	SF-12	SF-12 *Kiswahili version*	Low	Low	Low	High	NR	NR	High	NR	NR	NR	NR	NR
Not recommended	HIV QoL Scale-4 (Guo et al.)	HIV QoL Scale-4 (Guo et al.)	NR	NR	NR	High	Very low	NR	NR	NR	NR	NR	NR	NR
	HIV QoL Scale-2 (Meng et al.)	HIV QoL Scale-2 (Meng et al.)	NR	NR	NR	High	Very low	NR	High	High	High	NR	NR	NR
	HIV QoL Scale-3 (Su et al.)	HIV QoL Scale-3 (Su et al.)	Low	Low	Low	High	Very low	NR	NR	High	NR	NR	NR	NR
	HIV QoL Scal-1 (Xiang et al.)	HIV QoL Scale-1 (Xiang et al.)	Low	Low	Low	Moderate	Very low	Low	High	High	High	NR	NR	NR

EQ-5D, EuroQol-5 Dimensions; EUROHIS-QoL-8, European health interview surveys-quality of life-8; FAHI, Functional Assessment of HIV Infection; HAT-QoL, HIV/AIDS Targeted Quality of Life; HAQ-DI, Health Assessment Questionnaire Disability Index; HIV-QL31, HIV Disease Quality of Life 31-Item Instrument; HIV/AIDSQoL, HIV/AIDS Quality of Life Questionnaire; HOPES, HIV Overview of Problems Evaluation Scale; HUI3, Health Utility Index 3; ISSQoL, The Italian National Institute of Health Quality of Life; MQoL-HIV, Multidimensional Quality of Life Questionnaire for Persons with HIV/AIDS; MOS, Medical Outcomes Study; MOS-HIV, Medical Outcomes Study HIV Health Survey; MVQoLI, Missoula-Vitas Quality-of-Life Index; PROM, Patient-reported outcome measure; PROMIS, Patient-Reported Outcomes Measurement Information System; PROQoL-HIV, Patient-Reported Outcomes Quality of Life-HIV instrument; QWB, Quality of Well-Being scale; SF, Short Form Health Survey; WHOQoL, World Health Organization's Quality of Life; WHOQoL-BREF, The brief version of the World Health Organization's Quality of Life. WHOQoL-HIV, World Health Organization's Quality of Life Instrument in HIV Infection; WHOQoL-HIV-BREF, The brief version of the World Health Organization's Quality of Life Instrument in HIV Infection

## Discussion

This systematic review identified and assessed the psychometric properties of 30 HRQoL PROMs in PLWH and evaluated the certainty of the evidence provided for each PROM. To the best of our knowledge, this is the first and most comprehensive systematic review summarizing all psychometric properties of HRQoL PROMs for PLWH. The results may provide quantitative evidence for researchers and healthcare professionals to choose PROMs measuring HRQoL in PLWH in future scientific research and clinical practice.

Our systematic review found that compared to other HIV-specific and generic PROMs, the MOS-HIV has the best psychometric properties. The MOS-HIV is the most widely used HIV-specific instrument. In total, we searched fourteen validation studies to evaluate the psychometric properties of eight different language versions of MOS-HIV. Chinese included both simplified and traditional versions. Only one version was rated as “moderate” in internal consistency, and the other was rated as “high”. The MOS-HIV also has good construct validity, criterion validity, and hypothesis testing for construct validity. Overall, the expert group classified MOS-HIV as strongly recommended based on the GRADE system. Our results were in line with previous studies. Cooper and colleagues conducted umbrella reviews and found that the MOS-HIV was also recommended as a suitable measure for assessing HRQoL in PLWH from a content perspective [29]. In general, the MOS-HIV was considered to have good psychometric properties. Good internal consistency was generally reported, and its reliability was considered adequate [83, 84]. Acceptable convergent validity and discriminant validity were reported in several reviews [31, 32]. As one of the earliest HIV-specific HRQoL PROMs, MOS-HIV has been translated into at least 14 languages. The reliability and validity of the instrument were likely to decrease in the different translated versions due to their cultural adjustment. For these versions, mixed findings on the hypothesis testing of the MOS-HIV were reported [34–47]. As data on the psychometric properties of many studies were missing or indeterminate, we can draw only preliminary conclusions. More research is needed to fill the gap in the research on the psychometric properties of the existing instruments on HRQoL in PLWH.

Our review found that, in addition to MOS-HIV, the WHOQoL-HIV-BREF was reported to have good psychometric properties. Seven of eight different language versions of the WHOQoL-HIV-BREF were rated as “high” in hypothesis testing for construct validity. The WHOQoL-HIV-BREF was reported to have better reliability and internal consistency than other instruments except the MOS-HIV. Two language versions of the WHOQoL-HIV-BREF were rated as “very low” in internal consistency. Three language versions were rated as “very low”, and two were rated as “moderate” in construct validity. Connell and Skevington published a study to report the development and psychometric properties of the WHOQoL-HIV-BREF [51]. The results showed very good discriminant validity, which suggested the important role of the WHOQoL-HIV-BREF in distinguishing different stages of HIV disease progression [51].

Although the MOS-HIV showed good psychometric properties, a major advantage of the WHOQoL-HIV-BREF is its brevity. It contains only 31 items, and most participants can complete the instrument in 8 min. The WHOQoL-HIV-BREF is increasingly being used in HIV research. From a practical perspective, the MOS-HIV and WHOQoL-HIV-BREF focus on different dimensions and are based on different theoretical perspectives. The MOS-HIV is a multidimensional assessment measure that assesses physical, psychological, and social functioning. The MOS-HIV consists of 35 items across 11 domains: physical functioning, pain, social functioning, role functioning, emotional well-being, energy/fatigue, cognitive function, health distress, health transition, general health, and overall quality of life [8]. The WHOQoL-HIV-BREF has 31 items across six domains: physical functioning, psychological functioning, levels of independence, social relationships, environment, and spirituality [9].

The SF-36 is an internationally used generic instrument that can provide a comprehensive assessment of HRQoL in various populations. Although the SF-36 is also widely used in PLWH, only four validation studies were found in PLWH [19, 72–74]. The number of validation studies of different language versions was fewer than that of WHOQoL-HIV-BREF and MOS-HIV. From a global perspective, a better PROM should report decent psychometric properties in all language versions. Future studies are warranted to conduct validation studies evaluating the psychometric properties of the SF-36 in PLWH in various contexts. In addition, other aspects, such as scoring methods and content of items, may also restrict the wide usage of PLWH [85, 86]. Skevington et al. concluded that the SF-36 includes several different scoring scales and response options, which may complicate scoring and thus limit the widespread clinical use of the SF-36 [85]. Abbasi-Ghahramanloo et al. showed that the SF-36 may lack the ability to measure self-reported subjective HRQoL [86].

This study strongly recommends four HIV-specific and one generic PROM. Generic PROMs can be used to measure the HRQoL of general or HIV-infected populations. However, they may lack the sensitivity to detect subtle changes specific to PLWH, including stigma, relationship issues, and comorbidities [87]. HIV-specific PROMs are more closely related to the disease than generic PROMs and have the sensitivity and specificity needed for HIV-specific domains. Nonetheless, they are not conducive to use in comparisons across populations [88, 89]. It is highly recommended that when selecting instruments, researchers need to consider more aspects, including psychometric properties, instrument content coverage, ease of use, and scoring methods. Therefore, the choice of PROMs should be based on the specific aims of assessments and the response burden for participants.

Overall, we acknowledge that there are some limitations to this study. First, this study included only articles published in English or Chinese. Therefore, some studies published in other languages may not have been included, which may have affected the conclusions of this review. Second, we included only studies that aimed to evaluate the measurement properties of PROMs in PLWH. Some cross-sectional studies that aimed to explore the level of HRQoL in PLWH also reported the reliability and validity of PROMs. These types of studies were not included in this study. Third, we included four PROMs in Chinese that did not report a specific name. We used “unknown” to describe the names of these PROMs in all tables.

## Conclusions

This systematic review identified and described the psychometric properties of 30 instruments and 69 studies. The findings from the included studies highlighted that compared to other HIV-specific and generic HRQoL PROMs, the MOS-HIV had the best psychometric properties and could be recommended as the most suitable for use in research and clinics. We also strongly recommended using WHOQoL-HIV-BREF, SF-36, MQoL-HIV, and WHOQoL-HIV to evaluate HRQoL in PLWH. We suggest that the choice of PROMs should be based on the specific aims of assessments and the response burden for participants.

## Supplementary Information

**Additional file 1.** Searching Strategies and Results.

**Additional file 2.** PRISMA 2020 Checklist.

## Data Availability

Not applicable.

## Abbreviations

ART: Antiretroviral therapy
COSMIN: Consensus-based standards for the selection of health measurement instruments
EQ-5D: EuroQol-5 dimensions
EUROHIS-QoL-8: European health interview surveys-quality of life-8
FAHI: Functional assessment of HIV infection
HAT-QoL: HIV/AIDS targeted quality of life
GRADE: Grading, recommendations, assessment, development, and evaluation
HAQ-DI: Health Assessment Questionnaire Disability Index
HIV-QL31: HIV disease quality of life 31-item instrument
HIV/AIDSQoL: HIV/AIDS quality of life questionnaire
HOPES: HIV Overview of Problems Evaluation Scale
HRQoL: Health-related quality of life
HUI3: Health Utility Index 3
ISSQoL: The Italian National Institute of Health Quality of Life
MQoL-HIV: Multidimensional quality of life questionnaire for persons with HIV/AIDS
MOS: Medical outcomes study
MOS-HIV: Medical outcomes study HIV health survey
MVQoLI: Missoula-Vitas Quality-of-Life Index
PLWH: People living with HIV
PROM: Patient-reported outcome measure
PROMIS: Patient-reported outcomes measurement information system
PROQoL-HIV: Patient-reported outcomes quality of life-HIV instrument
QWB: Quality of well-being scale
SF: Short form health survey
WHOQoL: World Health Organization’s Quality of Life
WHOQoL-BREF: The brief version of the World Health Organization’s Quality of Life
WHOQoL-HIV: World Health Organization’s Quality of Life Instrument in HIV Infection
WHOQoL-HIV-BREF: The brief version of the World Health Organization’s Quality of Life Instrument in HIV Infection
