# Supplementary material for: Psychometric properties of self-reported measures of health-related quality of life in people living with HIV: a systematic review
Source: Health Qual Life Outcomes. 2022 Jan 10;20:5. doi: 10.1186/s12955-021-01910-w (PMC8744327; doi:10.1186/s12955-021-01910-w)
Supplement: Supplementary file 2 — Additional file 2. PRISMA 2020 Checklist. [file 12955_2021_1910_MOESM2_ESM.docx]

**Additional file 2**

**PubMed/Medline**

Search time: 2021-11-26 17:23

| **Search** | **Query** | **Items found** |
| --- | --- | --- |
| #1 | HIV Infections[MeSH] OR HIV[MeSH] OR Acquired Immunodeficiency Syndrome[MeSH] OR hiv[tiab] OR hiv infect*[tiab] OR AIDS[tiab] OR PLWHA[tiab] OR PLWH[tiab] OR human immunodeficiency virus[tiab] OR human immunedeficiency virus[tiab] OR human immuno‐deficiency virus[tiab] OR human immune‐deficiency virus[tiab] OR ((human immun*[tiab]) AND (deficiency virus[tiab])) OR acquired immunodeficiency syndrome[tiab] OR acquired immunedeficiency syndrome[tiab] OR acquired immuno‐deficiency syndrome[tiab] OR acquired immune‐deficiency syndrome[tiab] OR ((acquired immun*[tiab]) AND (deficiency syndrome[tiab])) Filters applied: from 1996/1/1 - 2020/5/1. | 346244 |
| #2 | “Quality of life”[MeSH] OR activities of daily living[Title/Abstract] OR anxiety[Title/Abstract] OR distress[Title/Abstract] OR hope[Text Word] OR quality of life[Text Word] OR QoL[Text Word] OR HRQoL[Title/Abstract] OR Well being[Title/Abstract] OR Hospital Anxiety and Depression Scale[Title/Abstract] Filters applied: from 1996/1/1 - 2020/5/1. | 629934 |
| #3 | Scale?[Title/Abstract] OR “patient reported outcome measur*”[Title/Abstract] OR PROM? [Title/Abstract] OR measure* [Title/Abstract] OR ”Patient Reported Outcome Measures*” [MeSH] OR “Surveys and Questionnaires” [MeSH] Filters applied: from 1996/1/1 - 2020/5/1. | 379059 |
| #4 | (instrumentation[sh] OR methods[sh] OR “Validation Study”[pt] OR “Comparative Study”[pt] OR “psychometrics”[MeSH] OR psychometr*[tiab] OR clinimetr*[tw] OR clinometr*[tw] OR "outcome assessment, health care"[MeSH] OR “outcome assessment”[tiab] OR “outcome measure*”[tw] OR “observer variation”[MeSH] OR “observer variation”[tiab] OR “Health Status Indicators”[Mesh] OR “reproducibility of results”[MeSH] OR reproducib*[tiab] OR “discriminant analysis”[MeSH] OR reliab*[tiab] OR unreliab*[tiab] OR valid*[tiab] OR “coefficient of variation”[tiab] OR coefficient[tiab] OR homogeneity[tiab] OR homogeneous[tiab] OR “internal consistency”[tiab] OR (cronbach*[tiab] AND (alpha[tiab] OR alphas[tiab])) OR (item[tiab] AND (correlation*[tiab] OR selection*[tiab] OR reduction*[tiab])) OR agreement[tw] OR precision[tw] OR imprecision[tw] OR “precise values”[tw] OR test-retest[tiab] OR (test[tiab] AND retest[tiab]) OR (reliab*[tiab] AND (test[tiab] OR retest[tiab])) OR stability[tiab] OR interrater[tiab] OR inter-rater[tiab] OR intrarater[tiab] OR intra-rater[tiab] OR intertester[tiab] OR inter-tester[tiab] OR intratester[tiab] OR intra-tester[tiab] OR interobserver[tiab] OR inter-observer[tiab] OR intraobserver[tiab] OR intra-observer[tiab] OR intertechnician[tiab] OR inter-technician[tiab] OR intratechnician[tiab] OR intra-technician[tiab] OR interexaminer[tiab] OR inter-examiner[tiab] OR intraexaminer[tiab] OR intra-examiner[tiab] OR interassay[tiab] OR inter-assay[tiab] OR intraassay[tiab] OR intra-assay[tiab] OR interindividual[tiab] OR inter-individual[tiab] OR intraindividual[tiab] OR intra-individual[tiab] OR interparticipant[tiab] OR inter-participant[tiab] OR intraparticipant[tiab] OR intra-participant[tiab] OR kappa[tiab] OR kappa’s[tiab] OR kappas[tiab] OR repeatab*[tw] OR ((replicab*[tw] OR repeated[tw]) AND (measure[tw] OR measures[tw] OR findings[tw] OR result[tw] OR results[tw] OR test[tw] OR tests[tw])) OR generaliza*[tiab] OR generalisa*[tiab] OR concordance[tiab] OR (intraclass[tiab] AND correlation*[tiab]) OR discriminative[tiab] OR “known group”[tiab] OR “factor analysis”[tiab] OR “factor analyses”[tiab] OR “factor structure”[tiab] OR “factor structures”[tiab] OR dimension*[tiab] OR subscale*[tiab] OR (multitrait[tiab] AND scaling[tiab] AND (analysis[tiab] OR analyses[tiab])) OR “item discriminant”[tiab] OR “interscale correlation*”[tiab] OR error[tiab] OR errors[tiab] OR “individual variability”[tiab] OR “interval variability”[tiab] OR “rate variability”[tiab] OR (variability[tiab] AND (analysis[tiab] OR values[tiab])) OR (uncertainty[tiab] AND (measurement[tiab] OR measuring[tiab])) OR “standard error of measurement”[tiab] OR sensitiv*[tiab] OR responsive*[tiab] OR (limit[tiab] AND detection[tiab]) OR “minimal detectable concentration”[tiab] OR interpretab*[tiab] OR ((minimal[tiab] OR minimally[tiab] OR clinical[tiab] OR clinically[tiab]) AND (important[tiab] OR significant[tiab] OR detectable[tiab]) AND (change[tiab] OR difference[tiab])) OR (small*[tiab] AND (real[tiab] OR detectable[tiab]) AND (change[tiab] OR difference[tiab])) OR “meaningful change”[tiab] OR “ceiling effect”[tiab] OR “floor effect”[tiab] OR “Item response model”[tiab] OR IRT[tiab] OR Rasch[tiab] OR “Differential item functioning”[tiab] OR DIF[tiab] OR “computer adaptive testing”[tiab] OR “item bank”[tiab] OR “cross-cultural equivalence”[tiab]) OR (“address”[Publication Type] OR “biography”[Publication Type] OR “case reports”[Publication Type] OR “comment”[Publication Type] OR “directory”[Publication Type] OR “editorial”[Publication Type] OR “festschrift”[Publication Type] OR “interview”[Publication Type] OR “lecture”[Publication Type] OR “legal case”[Publication Type] OR “legislation”[Publication Type] OR “letter”[Publication Type] OR “news”[Publication Type] OR “newspaper article”[Publication Type] OR “patient education handout”[Publication Type] OR “popular work”[Publication Type] OR “congress”[Publication Type] OR “consensus development conference”[Publication Type] OR “consensus development conference, nih”[Publication Type] OR “practice guideline”[Publication Type]) NOT (“animals”[MeSH Terms] NOT “humans”[MeSH Terms]) Filters applied: from 1996/1/1 - 2020/5/1. | 9049328 |
| #5 | #1 AND #2 AND #3 AND #4 | 3218 |

**Medline (Ovid)**

Search time: 2021-11-26 17:41

| **Search** | **Query** | **Items found** |
| --- | --- | --- |
| #1 | (HIV or Acquired Immunodeficiency Syndrome).sh. limit 1 to yr="1996 - 2020" | 42853 |
| #2 | (hiv or AIDS or PLWHA or PLWH or acquired immunodeficiency syndrome).ab,ti. limit 2 to yr="1996 - 2020" | 287221 |
| #3 | #1 OR #2 | 292902 |
| #4 | (Quality of life).sh. limit 4 to yr="1996 - 2020" | 198398 |
| #5 | (quality of life or QoL or HRQoL).ab,ti. limit 5 to yr="1996 - 2020" | 241227 |
| #6 | #4 OR #5 | 295253 |
| #7 | ("Patient Reported Outcome Measures*" or "Surveys and Questionnaires").sh. limit 7 to yr="1996 - 2020" | 459916 |
| #8 | (Scale? or PROM?).ab,ti. limit 8 to yr="1996 - 2020" | 635558 |
| #9 | #7 OR #8 | 1011469 |
| #10 | #3 AND #6 AND #9 | 1791 |

**EMBASE (Ovid)**

Search time: 2021-11-26 17:51

| **Search** | **Query** | **Items found** |
| --- | --- | --- |
| #1 | (HIV or HIV Infections or Acquired Immunodeficiency Syndrome).sh. limit 1 to yr="1996 - 2020" | 5493 |
| #2 | (hiv or AIDS or PLWHA or PLWH or acquired immunodeficiency syndrome).ab,ti. limit 2 to yr="1996 - 2020" | 414835 |
| #3 | #1 OR #2 | 415460 |
| #4 | (Quality of life).sh. limit 4 to yr="1996 - 2020" | 477255 |
| #5 | (quality of life or QoL or HRQoL).ab,ti. limit 5 to yr="1996 - 2020" | 457427 |
| #6 | #4 OR #5 | 594122 |
| #7 | ("Patient Reported Outcome Measure*" or "Surveys and Questionnaires").sh. limit 7 to yr="1996 - 2020" | 326 |
| #8 | (Scale? or PROM?).ab,ti. limit 8 to yr="1996 - 2020" | 1070961 |
| #9 | #7 OR #8 | 1071109 |
| #10 | #3 AND #6 AND #9 | 1429 |

**CINAHL (EBSCO)**

Search time: 2021-11-26 18:10

| **Search** | **Query** | **Items found** |
| --- | --- | --- |
| #1 | (MH HIV) OR (MH "Human Immunodeficiency Virus") OR (MH "Acquired Immunodeficiency Syndrome")Published Date: 19960101-20201231 | 15527 |
| #2 | (TI hiv OR AIDS OR PLWHA OR PLWH OR human immunodeficiency virus  OR human immuno‐deficiency virus OR human immunedeficiency virus OR human immune‐deficiency virus OR acquired immune‐deficiency syndrome OR acquired immunedeficiency syndrome OR acquired immunodeficiency syndrome OR acquired immuno‐deficiency syndrome)Published Date: 19960101-20201231 | 140187 |
| #3 | (AB hiv OR AIDS OR PLWHA OR PLWH OR human immunodeficiency virus  OR human immuno‐deficiency virus OR human immunedeficiency virus OR human immune‐deficiency virus OR acquired immune‐deficiency syndrome OR acquired immunedeficiency syndrome OR acquired immunodeficiency syndrome OR acquired immuno‐deficiency syndrome)Published Date: 19960101-20201231 | 140187 |
| #4 | #1 OR #2 OR #3 | 136117 |
| #5 | (MH "Quality of life")Published Date: 19960101-20201231 | 115675 |
| #6 | (TI activities of daily living OR quality of life OR QoL OR HRQoL)Published Date: 19960101-20201231 | 198713 |
| #7 | (AB activities of daily living OR quality of life OR QoL OR HRQoL)Published Date: 19960101-20201231 | 209544 |
| #8 | #5 OR #6 OR #7 | 198362 |
| #9 | (MH “Patient Reported Outcome Measures*” OR “Surveys and Questionnaires”)Published Date: 19960101-20201231 | 49 |
| #10 | (TI Scale? or "patient reported outcome measur*" or PROM? or measure*)Published Date: 19960101-20201231 | 829046 |
| #11 | (AB Scale? or "patient reported outcome measur*" or PROM? or measure*)Published Date: 19960101-20201231 | 928747 |
| #12 | #9 OR #10 OR #11 | 891198 |
| #13 | #4 AND #8 AND #12 | 1533 |

**Web of Science**

Search time: 2021-11-26 18:55

| **Search** | **Query** | **Items found** |
| --- | --- | --- |
| #1 | TOPIC: (HIV OR Acquired Immunodeficiency Syndrome OR AIDS OR PLWHA OR PLWH OR human immunodeficiency virus  OR human immuno‐deficiency virus OR human immunedeficiency virus OR human immune‐deficiency virus OR acquired immune‐deficiency syndrome OR acquired immunedeficiency syndrome OR acquired immunodeficiency syndrome OR acquired immuno‐deficiency syndrome) Timespan: 1996-2020. | 78566 |
| #2 | TOPIC: (activities of daily living OR quality of life OR QoL OR HRQoL OR Hospital Anxiety and Depression Scale ) Timespan: 1996-2020. | 67987 |
| #3 | TOPIC: (“Patient Reported Outcome Measures*” OR “Surveys and Questionnaires” OR Scale? OR PROM? or measure*) Timespan: 1996-2020. | 411808 |
| #4 | #1 AND #2 AND #3 | 1291 |

**ProQuest Dissertations and Theses (Health & Medical Collection)**

Search time: 2021-11-26 19:04

| **Search** | **Query** | **Items found** |
| --- | --- | --- |
| #1 | mainsubject(HIV OR Acquired Immunodeficiency Syndrome ) AND pd(19960101-20200501) | 116999 |
| #2 | ti(hiv OR AIDS OR PLWHA OR PLWH OR acquired immunedeficiency syndrome) AND pd(19960101-20200501) | 119177 |
| #3 | ab(hiv OR AIDS OR PLWHA OR PLWH OR acquired immunedeficiency syndrome) AND pd(19960101-20200501) | 199117 |
| #4 | #1 OR #2 OR #3 | 250176 |
| #5 | mainsubject(Quality of life) AND pd(19960101-20200501) | 101568 |
| #6 | ti(activities of daily living OR QoL OR HRQoL) AND pd(19960101-20200501) | 2498 |
| #7 | ab(activities of daily living OR QoL OR HRQoL) AND pd(19960101-20200501) | 39596 |
| #8 | #5 OR #6 OR #7 | 122941 |
| #9 | mainsubject(Patient Reported Outcome Measure*) OR mainsubject(Surveys AND Questionnaires) AND pd(19960101-20200501) | 9454 |
| #10 | ti(Scale? OR PROM? or measure*) AND pd(19960101-20200501) | 114900 |
| #11 | ab(Scale? OR PROM? or measure*) AND pd(19960101-20200501) | 1199531 |
| #12 | #9 OR #10 OR #11 | 1246920 |
| #13 | #4 AND #8 AND #12 | 1438 |

**Cochrane Library**

Search time: 2021-11-26 21:30

| **Search** | **Query** | **Items found** |
| --- | --- | --- |
| #1 | HIV Infections OR HIV OR Human Immunodeficiency Virus OR Acquired Immunodeficiency Syndrome OR AIDS OR PLWHA OR PLWH OR human immunodeficiency virus OR acquired immunedeficiency syndrome OR acquired immunodeficiency syndrome OR acquired immuno-deficiency syndrome in Title Abstract Keyword - with Cochrane Library publication date Between Jan 1996 and May 2020 (Word variations have been searched | 30382 |
| #2 | Quality of life OR activities of daily living OR QoL OR HRQoL in Title Abstract Keyword - with Cochrane Library publication date Between Jan 1996 and May 2020 (Word variations have been searched | 117660 |
| #3 | “Patient Reported Outcome Measures*” OR “Surveys and Questionnaires” OR Scale? OR PROM? in Title Abstract Keyword - with Cochrane Library publication date Between Jan 1996 and May 2020 (Word variations have been searched | 199347 |
| #4 | “addresses” OR “biography” OR “case reports” OR “comment” OR “directory” OR “editorial” OR “festschrift” OR “interview” OR “lectures” OR “legal cases” OR “legislation” OR “letter” OR “news” OR “newspaper article” OR “patient education handout” OR “popular works” OR “congresses” OR “consensus development conference” OR “consensus development conference, nih” OR “practice guideline” NOT (“animals” NOT “humans” ) in Title Abstract Keyword - with Cochrane Library publication date Between Jan 1996 and May 2020 (Word variations have been searched | 49677 |
| #5 | #1 AND #2 AND #3 AND #4 | 71 |

CNKI

Search time: 2020-10-24 11:30

| **Search** | **Query** | **Items found** |
| --- | --- | --- |
| #1 | [篇关摘%人类免疫缺陷病毒 + 获得性免疫缺陷综合征 + 艾滋病 + HIV + AIDS](https://kns.cnki.net/KNS8/AdvSearch?id=54&dbcode=SCDB&searchtype=gradeSearch&ishistory=1) | 111900 |
| #2 | 篇关摘%生活质量 + QoL +日常活动 + 生活活动 + 焦虑 + 痛苦 + 希望 + HRQoL + 健康感知 + 焦虑和抑郁量表 + 症状困扰量表 | 1515200 |
| #3 | 篇关摘%患者报告结局 + 量表 + 问卷 | 1002100 |
| #4 | #1 AND #2 AND #3 | 1094 |

WanFang

Search time: 2020-10-24 11:50

| **Search** | **Query** | **Items found** |
| --- | --- | --- |
| #1 | (主题:(HIV OR AIDS OR 人类免疫缺陷病毒 OR 获得性免疫缺陷综合征 OR 艾滋病))*Date:1996-2020 | 406293 |
| #2 | (主题:(生活质量 OR QoL OR日常活动 OR 生活活动 OR 焦虑 OR 痛苦 OR 希望 OR HRQoL OR 健康感知 OR 焦虑和抑郁量表 OR 症状困扰量表))*Date:1996-2020 | 2590699 |
| #3 | (主题:(患者报告结局 OR 量表 OR 问卷))*Date:1996-2020 | 1054295 |
| #4 | #1 AND #2 AND #3 | 1506 |
